# ISCEV extended protocol for the photopic On–Off ERG

**DOI:** 10.1007/s10633-018-9645-y

**Published:** 2018-06-22

**Authors:** Maja Sustar, Graham E. Holder, Jan Kremers, Claire S. Barnes, Bo Lei, Naheed W. Khan, Anthony G. Robson

**Affiliations:** 1 Eye Hospital, University Medical Centre Ljubljana, Ljubljana, Slovenia; 2 Department of Electrophysiology, Moorfields Eye Hospital, London, UK; 3 Department of Ophthalmology, National University of Singapore, Singapore, Singapore; 4 University of Sydney Medical School, Sydney, Australia; 5 Institute of Ophthalmology, University College London, London, UK; 6 Department of Ophthalmology, University Hospital Erlangen, Erlangen, Germany; 7 Palo Alto, USA; 8 Henan Eye Institute, Henan Eye Hospital, Zhengzhou, China; 9 Department of Ophthalmology and Visual Science, University of Michigan, Ann Arbor, USA

**Keywords:** Clinical standards, Electroretinogram (ERG), Full-field ERG, International Society of Clinical Electrophysiology of Vision (ISCEV), On–Off ERG, Long-duration ERG, Bipolar cells, Retinopathy, Retinal dystrophy

## Abstract

The International Society for Clinical Electrophysiology of Vision (ISCEV) standard for full-field electroretinography (ERG) describes a minimum procedure, but encourages more extensive testing. This ISCEV extended protocol describes an extension to the ERG standard, namely the photopic On–Off ERG, and outlines common clinical applications. A light stimulus duration of 150–200 ms is used in the presence of a rod-suppressing background to elicit cone-driven On- and Off-system ERG components. The On-response occurs after the stimulus onset and has a negative a-wave and positive b-wave. The Off d-wave is a positive component evoked by stimulus offset. Common diagnoses that may benefit from additional photopic On–Off ERG testing include retinal dystrophies and retinal disorders that cause dysfunction at a level that is post-phototransduction or post-receptoral. On–Off ERGs assess the relative involvement of On- and Off-systems and may be of use in the diagnosis of disorders such as complete and incomplete congenital stationary night blindness (complete and incomplete CSNB), melanoma-associated retinopathy, and some forms of autoimmune retinopathy. The photopic On–Off ERGs may also be useful in X-linked retinoschisis, Batten disease, Duchenne muscular dystrophy, spinocerebellar degeneration, quinine toxicity, and other retinal disorders.

## Introduction

The International Society for Clinical Electrophysiology of Vision (ISCEV) standard for full-field electroretinography (ERG) describes a minimum set of tests but encourages the use of additional ERG protocols for clinical ERG testing [1]. This extended protocol describes the photopic On–Off ERG, as a specialized procedure which is well established and broadly accepted by experts in the field. The protocol was prepared by the authors in accordance with ISCEV procedures http://www.iscev.org/standards/index.html), and was approved by the ISCEV Board of Directors on 25th March 2018.

## Scope and applications

The standard light-adapted (LA) 3.0 ERG b-wave is evoked with flashes of a short duration (< 5 ms) on a rod-saturating background and largely reflects overlapping contributions from the On- and Off-bipolar cell pathways. Separation of the function of the On- and Off-pathways requires long-duration stimuli (e.g., 150–200 ms) in the presence of a rod-saturating background. The photopic On–Off ERG has two major components, the On-response and the Off-response. The On-response occurs after the stimulus onset and consists of two prominent waves, the negative polarity a-wave and the positive b-wave. The Off-response, or d-wave, is a positive polarity component in response to stimulus offset [2–4]. The sources of On- and Off-responses were elucidated by experimental pharmacological studies in non-human primates, whose ERGs are very similar to those in humans. These studies showed that the a-wave of the On-response originates from cone photoreceptors, with a significant contribution from Off- (hyperpolarizing) bipolar cells [5]. The b-wave of the On-response reflects the function of the On- (depolarizing) bipolar cells, although its amplitude and shape may be influenced by Off-bipolar and horizontal cells [6]. The d-wave is a complex response; the initial rapid phase originates from Off-bipolar cell activity, but cone photoreceptors contribute to the later slow phase and On-bipolar cells act in an opposite polarity direction [7, 8].

Common diagnoses that may benefit from additional photopic On–Off ERG testing include retinal dystrophies and retinal disorders that cause dysfunction post-phototransduction or at a post-receptoral level. The photopic On–Off ERG allows evaluation of the relative or selective involvement of On- and Off-pathways, not fully enabled by the standard LA 3.0 ERG responses [2–4, 9–15]. Common forms of congenital stationary night blindness (CSNB) are good illustrative examples. In complete CSNB, there is generalized On-bipolar cell dysfunction, and the waveform shows an electronegative On-response but a preserved Off-response. In contrast, incomplete CSNB is associated with abnormalities affecting both the On- and Off-responses [2]. Other retinal disorders associated with selective On-pathway dysfunction include melanoma-associated retinopathy, early cases of phosphomannomutase deficiency (PMM2-CDG) [12], and some forms of autoimmune retinopathy [15]. The photopic On–Off ERGs may also be useful in X-linked retinoschisis, Batten disease, Duchenne muscular dystrophy, spinocerebellar degeneration, quinine toxicity, and other disorders [16, 17].

## Patient population

Patients of all ages able to tolerate Ganzfeld stimulation, referred for investigation of possible retinal dysfunction, especially those with decreased light-adapted and/or dark-adapted ERG b-wave and relatively preserved a-wave (electronegative ERG or low b:a ratio), suggesting dysfunction post-phototransduction or at the level of the bipolar cells.

## Technical issues

The photopic On–Off ERG protocol will follow the specifications of the current ISCEV standard ERG [1]. Additional considerations include the following:

(a) *Stimulus duration*. The duration of the light stimulus should be long enough to separate the On- and Off-responses. Most studies used durations of between 150 and 200 ms (Table 1 in appendix), based on the largest d-wave amplitude attained, although statistically, smaller amplitudes are reported only for flash durations shorter than 75 ms [18]. Further increase in the d-wave amplitude was observed with stimuli up to 900 ms duration [19], but responses to such long durations take longer to record. Patient comfort and minimizing possible eye closure and blink artefacts are considerations when selecting the light duration.

(b) *Stimulus wavelength*. The flash and background wavelength for the ISCEV standard ERG are defined as visibly white, with CIE coordinates near *x* = 0.31, *y* = 0.32. Both white and chromatic (blue and green) stimuli have been used to elicit On–Off ERGs of similar waveform [18]. Some laboratories use orange stimuli in the presence of green background, for more selective stimulation of L-and M-cone system with simultaneous suppression of the rods and S-cones [17, 20]. These stimuli are effective at eliciting On- and Off-responses and have been shown to be informative in numerous studies. Longer wavelength stimuli (red) may decrease the d-wave and change the shape of the b-wave [18, 19] and should be avoided.

(c) *Stimulus strength and background luminance*. Brighter backgrounds require stronger stimuli to elicit detectable responses [18]. If stimuli are too weak, responses are small. If stimuli are too strong the b-wave becomes broader and peak time variable and difficult to determine [18], while the d-wave becomes either decreased or dominated by a component of longer peak time (the basis of the photopic hill phenomenon) [21]. Strong stimuli and backgrounds may also be poorly tolerated by some patients.

## Calibration

The protocol is technically similar to that for the ISCEV standard ERG, and the calibration and frequency of calibration should follow the latest ISCEV standard [1]. The strength of the stimulus and background luminance should be specified in photopic candelas per meter squared (phot cd m^−2^).

## Protocol specification

Patient preparation follows that for the current ISCEV standard ERG [1]. It is suggested that for routine applications the photopic On–Off ERG is added to the ISCEV standard protocol after the other LA ERGs. This extended protocol has the following additional specifications.

(a) *Stimulus duration*. A stimulus duration of 150 ms or 200 ms. To allow clear separation of On- and Off- responses, efficient signal averaging and to maintain consistency with the majority of published clinical studies to date.

(b) *Stimulus wavelength*. A white stimulus on a white background (as used in the majority of published studies) or a chromatic light on a chromatic background (to suppress rods and S-cones) may be used providing longer wavelength (red) stimuli are avoided. Orange (620 nm) stimuli in the presence of green (560 nm) background may allow more selective stimulation of L-and M-cone systems with simultaneous suppression of the rods and S-cones.

(c) *Stimulus strength and background luminance*. Stimulus luminance for white stimuli is 250 cd m^−2^ or within the range phot 150–350 cd m^−2^ with a background luminance of 30 cd m^−2^.

(d) *Frequency of stimulus presentation*. A stimulus rate of ≤ 2 per second (inter-stimulus interval ≥ 0.5 s), as for the current ISCEV Standard LA 3.0 ERG.

## Response evaluation

**Fig. 1:**
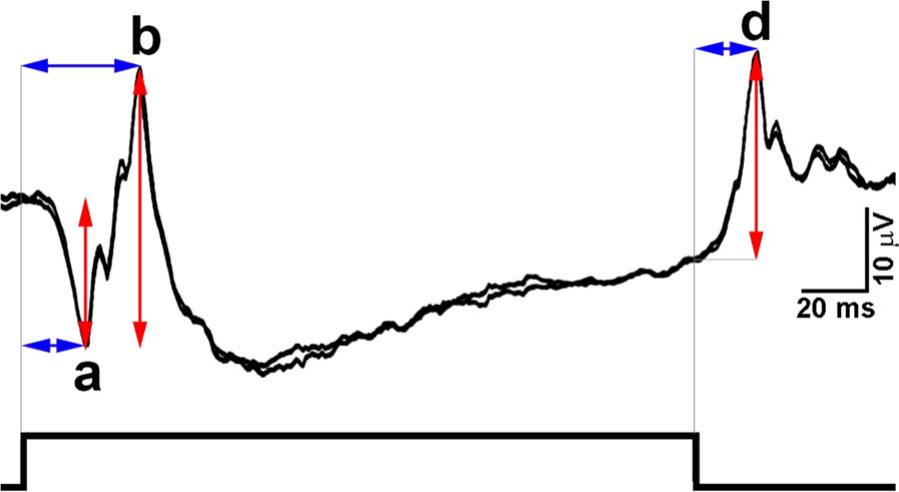
Diagram of the three main components of the photopic On–Off ERG and their measurement. Stimulus duration is indicated at the bottom of the figure; red (vertical) arrows indicate amplitude measurement, blue (horizontal) arrows indicate peak time measurement. Two traces are superimposed to demonstrate reproducibility

The photopic On–Off ERG response consists of three prominent waves, the a- and b-waves as part of the On-response and the Off-response complex, mainly the d-wave (Fig. 1). A negative a-wave appears in response to the onset of the stimulus, followed by the positive-going On-response b-wave. The d-wave of the Off-response is the positive peak, which occurs after stimulus offset. The amplitude of the a-wave is measured from the baseline to the first negative trough. The amplitude of the b-wave is measured from the trough of the a-wave to the peak of the b-wave. The amplitude of the d-wave is measured from the time point of stimulus offset to the peak of the d-wave. Peak times of On-response components are measured from the beginning of the stimulus to the trough of the a-wave and peak of the b-wave; Off-response peak time is measured from stimulus offset to the peak of the d-wave. The amplitude and peak time values should be evaluated and compared with reference normative data, established in each laboratory for its own equipment, recording protocols and patient population.

## Reporting

This protocol is intended to be used for routine applications as an extension to the standard ERG protocol, and reporting should follow the latest ISCEV standard for ERG [1]. Additionally, the spectral characteristics of the stimulus and background should be acknowledged if different from the standard LA ERG (e.g., peak wavelength and bandwidth). The duration of the light stimulus should be stated and luminance (in phot cd m^−2^) of stimulus and background should be stated. Unless already embedded within the ISCEV standard ERG protocol, pupil size and duration of light adaption should be stated. The amplitude of the a-, b-, and d-waves and respective time to peaks may be reported along with age-appropriate laboratory reference normative data. It is acknowledged that in diagnostic studies involving ISCEV standard ERGs, it may be sufficiently informative to describe the relative involvement of On–Off ERG a-, b-, and d-waves qualitatively.

## Appendix

**Experimental procedures excluded from this extended protocol**

ERG recordings with long-duration stimuli can be affected by technical issues, and blink and squint artefacts may disturb the recordings. Some authors suggest that this may be alleviated by using stimuli with sawtooth luminance profiles. The recordings are typically performed at photopic luminances. Instead of a stimulus upon a background, these stimuli are modulated around a mean. The stimulus strength is quantified by Michelson contrast (*C*): *C* = (*L*_max_ + *L*_min_)/(*L*_max_ − *L*_min_) in which *L*_max_ and *L*_min_ are the maximal and minimal luminance in the stimulus. Since this stimulus is given repetitively with a frequency between 2 and 8 Hz, blink artefacts do not play a large role. Higher frequencies are not recommended because the responses to subsequent stimuli may merge. On- and Off-responses are obtained separately by using rapid-ON and rapid-OFF stimuli. They have been used in a variety of disorders [22–27] and may have benefits, but have not been widely available on commercial systems. On- and Off-responses may not be the same for all stimulus types. It has been reported that On- and Off-responses originating in the L-cones have the same morphology as those obtained with luminance stimuli. In contrast, M-cone-driven On-responses resemble Off-responses with L-cone isolating and luminance stimuli and vice versa, suggesting that cone opponent processes may be involved [28–30]. Beside the sawtooth stimulation, increment and decrement stimulation is another way of eliciting the d-wave without a major impact of blink artefacts [31].

An alternative method extracts On- and Off-responses from the LA 3.0 ERG through the quantification of wavelet coefficients by discrete wavelet transform (DWT) analyses [32]. This approach suggests the activity of the retinal On-pathway to be related to a 20 Hz component of the photopic ERG, while the Off-pathway activity is reflected by a 40-Hz component. This finding was based on the fact that 20 and 40 Hz components of the photopic b-wave are selectively attenuated in case of imbalanced dysfunction of the On- and Off-pathways in some diseases [32], confirmed with the DWT of the photopic On–Off ERGs [33].

Since the photopic On–Off ERG is potentially valuable ERG method in animal studies, researchers should be aware that positive On- and Off-responses, as those in humans, can only be recorded in some non-human primates, while On- and Off-responses with electronegative waveform are present in rodents including mice and rats [34].

### Justification for the protocol details

A literature review was performed with Medline search engine to find publications that reported photopic On–Off ERG using following keywords: electroretinogram or full-field ERG or long-duration flash, b-wave or ON-response, d-wave or OFF-response. Studies from the year 1986 to 2016 were reviewed and those that specified stimulus parameters are summarized in Table 1. For the purposes of developing a clinical protocol animal studies were excluded, as were studies using multifocal, focal and saw tooth type of stimulation and studies focusing on the function of rod or ganglion cell systems. Other exclusions included responses obtained by silent substitution [28–30] and analyses of On and Off components derived from discrete wavelet transform (DWT) analyses of the LA 3 ERG [32, 33]. Studies from the year 1986 to 2016 were reviewed and those that specified stimulus parameters are summarized in Table 1.

**Table 1:** Previously published recording parameters

References	Stimulus strength	Background luminance	Stimulus duration (ms)	Stimulus and background wavelength
[4]	185 cd m^−2^	43 cd m^−2^	150	White stimulus on white background
[3]	3.7 log cd m^−2^ (5011 cd m^−2^)	2.1 log cd m^−2^ (125 cd m^−2^)	5–100	White stimulus on white background
[9, 53]	3 cd s m^−2^ (15 cd m^−2^)^a^	10 cd m^−2^	200 and 250	Red and green stimulus on white background
[10]	750 cd m^−2^	42 cd m^−2^	500	White stimulus on white background
[11]	3 cd s m^−2^ (12 cd m^−2^)	10 cd m^−2^	256	630 nm stimulus on white background
[12]	200 cd m^−2^	43 cd m^−2^	90, 120	White 6500K
[13]	3 log cd s m^−2^ (5000 cd m^−2^)	2 log cd m^−2^ (100 cd m^−2^)	200	White stimulus on white background
[14]	398 cd m^−2^	48 cd m^−2^	200	White stimulus on white background
[21]	0.6–3.5 log cd m^−2^ (4–3162 cd m^−2^)	40 cd m^−2^	250	White stimulus on white background
[18, 56]	0.4–2.1 log cd s m^−2^ (12.5–629 cd m^−2^)	20-50 cd m^−2^	5–200	White, 460, 508 and 667 nm stimulus on white background
[15, 17, 20, 38, 41, 52]	560 cd m^−2^	160 cd m^−2^	150–200	620 nm stimulus on 530 nm background
[35]	440 cd m^−2^	160 cd m^−2^	200	612 nm stimulus on 530 nm background
[36]	133 cd m^−2^	43 cd m^−2^	120	White 6500K
[37]	1.7 log cd s m^−2^ (250 cd m^−2^)	40 cd m^−2^	200	White stimulus (6500K) on white background
[39]	1 cd s/m^2^ (4 cd m^−2^)	30 cd m^−2^	250	White 6500K
[40]	200 cd m^−2^	42 cd m^−2^	150	White stimulus on white background
[42]	4 log ph td (200 cd m^−2^)^b^	3.3 log ph td (40 cd m^−2^)^b^	150	White stimulus on white background
[43]	200 cd m^−2^	30 cd m^−2^	100	White stimulus on white background
[44]	225 cd m^−2^	30 cd m^−2^	188	White stimulus on white background
[45–48]	300 cd m^−2^	40 cd m^−2^	150	White stimulus on white background
[49]	650 cd m^−2^	160 cd m^−2^	120, 200	Orange stimulus on green background
[50]	200 cd m^−2^	42 cd m^−2^	150	White stimulus on white background
[51]	1700 cd m^−2^	28 cd m^−2^	125	White stimulus on white background
[54]	360 cd m^−2^	40 cd m^−2^	100	White stimulus on white background
[55, 57]	1120 cd m^−2^	30 cd m^−2^	200	White stimulus on white background
[58]	40,60,80 cd m^−2^	20 cd m^−2^	240	White stimulus on white background
[59]	2.5 log cd m^−2^ (316 cd m^−2^)	40 cd m^−2^	150	White stimulus on white background

^a^Time integrated luminance divided by the length of the stimulus in seconds

^b^Calculated for 8 mm pupil diameter
